# The Effect of Particle Shell on Cooling Rates in Oil-in-Oil Magnetic Pickering Emulsions

**DOI:** 10.3390/ma13214783

**Published:** 2020-10-26

**Authors:** Rafał Bielas, Arkadiusz Józefczak

**Affiliations:** Chair of Acoustics, Faculty of Physics, Adam Mickiewicz University in Poznań, Uniwersytetu Poznańskiego 2, 61-614 Poznań, Poland

**Keywords:** Pickering emulsion, particle-stabilized emulsion, magnetic heating, magnetic field, coalescence, calorimetric measurements

## Abstract

Pickering emulsions (particle-stabilized emulsions) are usually considered because of their unique properties compared to surfactant-stabilized emulsions including better stability against emulsion aging. However, the interesting feature of particle-stabilized emulsions could be revealed during their magnetic heating. When magnetic particles constitute a shell around droplets and the sample is placed in an alternating magnetic field, a temperature increase appears due to energy dissipation from magnetic relaxation and hysteresis within magnetic particles. We hypothesize that the solidity of the magnetic particle shell around droplets can influence the process of heat transfer from inside the droplet to the surrounding medium. In this way, particle-stabilized emulsions can be considered as materials with changeable heat transfer. We investigated macroscopically heating and cooling of oil-in-oil magnetic Pickering emulsions with merely packed particle layers and these with a stable particle shell. The change in stability of the shell was obtained here by using the coalescence of droplets under the electric field. The results from calorimetric measurements show that the presence of a stable particle shell caused a slower temperature decrease in samples, especially for lower intensities of the magnetic field. The retarded heat transfer from magnetic Pickering droplets can be utilized in further potential applications where delayed heat transfer is desirable.

## 1. Introduction

Emulsions are ubiquitous systems utilized in a very broad range of industrial branches, in medicine, and in our daily life. In the last decades, emulsions stabilized with surfactants have been gradually replaced by Pickering emulsions, i.e., emulsions stabilized with particles rather than surface-active chemicals. Such systems are indicated as more eco-friendly promising materials for food processing [1,2], industrial applications such as oil recovery [3,4] or protection of wood [5], and formulation of new pharmaceutics [6,7]. The unique properties of particles used as stabilizers open new opportunities for practical use, such as a controlled release of cargo encapsulated in droplets under external stimuli. In this context, magnetic Pickering emulsions are often invoked. The shell around emulsion droplets consisting of materials susceptible to the magnetic field can result in the controlled positioning of droplets [8]. The alternating magnetic field can induce relaxation processes (namely Néel and Brown) or magnetic hysteresis in magnetic particles that lead to losses of magnetic energy [9]. This energy dissipation is converted into heat, which is a well-known fact utilized, among others, in magnetic hyperthermia therapies [10,11] and magnetically-induced catalysis to perform reactions such as CO_2_ methanation [12]. This fact also makes the heating generated in magnetic Pickering emulsions the potential object of interest.

In Pickering emulsions, particles adsorb to the droplet surface due to capillary forces. The solid particle shell formed in such a way prevents coalescence of the droplets and Oswald ripening [13]. However, when magnetic particles are used as stabilizers, their presence alters not only the stability of emulsion droplets. During the application of the AC magnetic field, every particle is a source of heat. The fact that they reside in the interface between the droplet and surrounding medium results in heating both the inside of the droplet and the layer around the droplet. The heat transfer from the droplet inside might be somehow retarded, especially for the high-temperature increase, because of solidity of the shell. This can influence the therapeutic effect when Pickering droplets would be used in magnetic hyperthermia, but it can also open new applications of this material, that is, the material having a heat transfer latency.

It is well-known that particles can enhance the properties of liquids serving as coolants e.g., in transformers. Additionally, magnetic particles were used to enrich cooling transformer oils because they may improve thermal conductivity [14], so the elements of the transformer can be better protected against over-heating. In the case of Pickering droplets capsulated in a solid magnetic particle shell, the situation can be opposite as the heat transfer through the emulsion with droplets coated by magnetic particles and immersed in surrounding liquid is hindered. Potentially, the heat entrapped within the droplet could be transported and released under external stimuli in the destined place. To prove the existence of the aforementioned phenomenon, the comparison between emulsion systems in two different stages, i.e., one with droplets poorly coated with magnetite particles and another with a reinforced particle shell, was performed. The use of a two-step approach to fabricating Pickering emulsions by using ultrasound homogenization and an electric field [15] gives us the unique opportunity to test droplets with varying degrees of coverage by solid particles. As we will show, this difference also influenced also the process of heat transfer that is reflected in different cooling rates.

To investigate the process of cooling in a quantitative way the so-called Newton’s law can be used [16]. In the case of our study, the temporal temperature measured in the emulsion system after the alternating magnetic field is turned off can be expressed as [17]:

(1) $$T\left(t\right)=T_{sur}+\left(T_{0}-T_{sur}\right)exp\left(-\frac{t}{\tau }\right),$$

where $T_{sur}$ refers to the temperature of the medium surrounding the sample, $T_{0}$ is the temperature at the beginning of the cooling process and τ is a derived parameter, the cooling time constant that can be considered as a measure of cooling rate. Another approach is also possible. Usually, to evaluate the heating performance of a given medium, non-adiabatic setups are used. This is because the measurements using adiabatic systems are time-consuming, expensive, and hard to construct. However, the measurements under non-adiabatic conditions can be still be considered as reliable. They are very common but, some reports suggest the strong need of including quantified non-adiabatic losses into the calculation of the final results [18]. Under the non-adiabatic conditions of calorimetric measurements commonly represented in scientific papers, the loss of provided external energy from the sample to the environment starts when the temperature of the sample exceeds the temperature of the surrounding medium. This loss is due to three main mechanisms, i.e., thermal conduction, convection, and radiation. Because the effect of those mechanisms is dependent on the temperature gradient inside the sample to varying degrees, the non-linear relation between the temperature *T* and power loss can be expressed as [18,19]:

(2) $$c_{p}\cdot \frac{dT\left(t\right)}{dt}=P+L\Delta T+B\Delta T^{2}+C\Delta T^{3}+R\Delta T^{4}$$

where $c_{p}$ is the specific heat of the sample, P is the power provided in the process of heating, and the parameters *L*, *B*, *C*, and *R* are coefficients in a fourth-order polynomial. The order of the polynomial is motivated by the mechanism of thermal radiation that depends on the fourth power of temperature difference and certainly occurs. According to [18,19], for small differences between the temperature of the surrounding medium and the temperature of the sample, it should be possible to simplify the abovementioned expression to the linear relation.

In this work, we performed the calorimetric measurements under non-adiabatic conditions on two types of magnetic emulsion systems: the first system formed by using ultrasonic homogenization with poor particle coating around the droplets and the second system formed by ultrasonic homogenization and subsequent electro-coalescence that resulted in much better coverage of the droplets. The measurements provided evidence showing that a difference in the solidity of magnetic particle shells around emulsion droplets influenced not only heating but also cooling rates.

## 2. Materials and Methods

### 2.1. Particles and Oils

Three types of magnetic particles (MPs) were utilized in our experiments as stabilizers in magnetite-stabilized emulsions: magnetic microparticles with declared sizes of <5 µm (µMPs) purchased from Sigma-Aldrich, magnetic nanoparticles with sizes of 50–100 nm (nMPs) purchased from Sigma-Aldrich and magnetic nanoparticles with sizes of 10 nm (OA-MPs) synthesized in the process of co-precipitation and additionally functionalized with oleic acid as a surfactant as described in [20]. We prepared oil-in-oil emulsions where castor oil (MERLIN, MA 220-1) was the continuous phase and silicone oil (Rhodorsil oils 47 V 50) was the dispersed phase. The basic characteristics of the materials used are presented in Table 1.

### 2.2. Ultrasound and the Electric Field in the Formation of Magnetic Pre-emulsions and Emulsions

**Figure 1:**
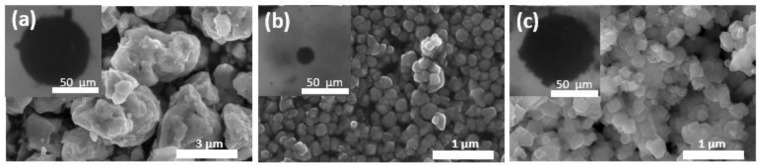
Scanning electron microscopy data for (**a**) OA-MPs, (**b**) nMPs, and (**c**) µMPs. The inset images present examples of emulsion droplets coated by these particles after stabilization in an electric field.

The process of formation of the colloidal systems tested in this experiment was done based on a two-step procedure involving ultrasonic homogenization and coalescence in an electric field first developed in [15]. Briefly, an ultrasonic homogenizer (Sonoplus HD 300, Bandelin, Berlin, Germany, working frequency of 18 kHz) was used for the sonication of samples with a fixed concentration of substrates, i.e., 10% *w*/*w* of silicone oil and 2.3% w/w of magnetite particles. The sonication time was 60 s or 120 s for systems with non-functionalized particles and particles with oleic acid coating, respectively. The acoustic intensity was estimated as 17 W/cm^2^. As shown in our previous articles [15,23], for oil-in-oil emulsions ultrasonic homogenization resulted in the formation of small droplets barely coated by particles that we consistently called the pre-emulsion. An electric field was utilized to stabilize these droplets via consecutive events of coalescence. As a result, after 20 min under the electric field of 200–600 V/mm the coverage of droplets by particles increased significantly to the level that prevented their further coalescence. The results from SEM (scanning electron microscopy imaging) for particles used as stabilizers are presented in Figure 1. The examples of stable Pickering droplets taken by optical microscopy were also included as inset pictures.

The process of Pickering emulsion formation under the electric field is governed by the so-called limited coalescence regime [15]. The final droplet size depends on the size of particles and their concentration in relation to the dispersed phase. As we mentioned above, the concentrations of both silicone oil and particles were fixed. This means that the size of fully covered Pickering droplets should be related to the size of magnetic particles used as stabilizers. This is not fulfilled in the case of emulsions with small particles functionalized with oleic acid (OA-MPs), which is caused by the presence of a surfactant layer.

### 2.3. Idea of the Experiment

**Figure 2:**
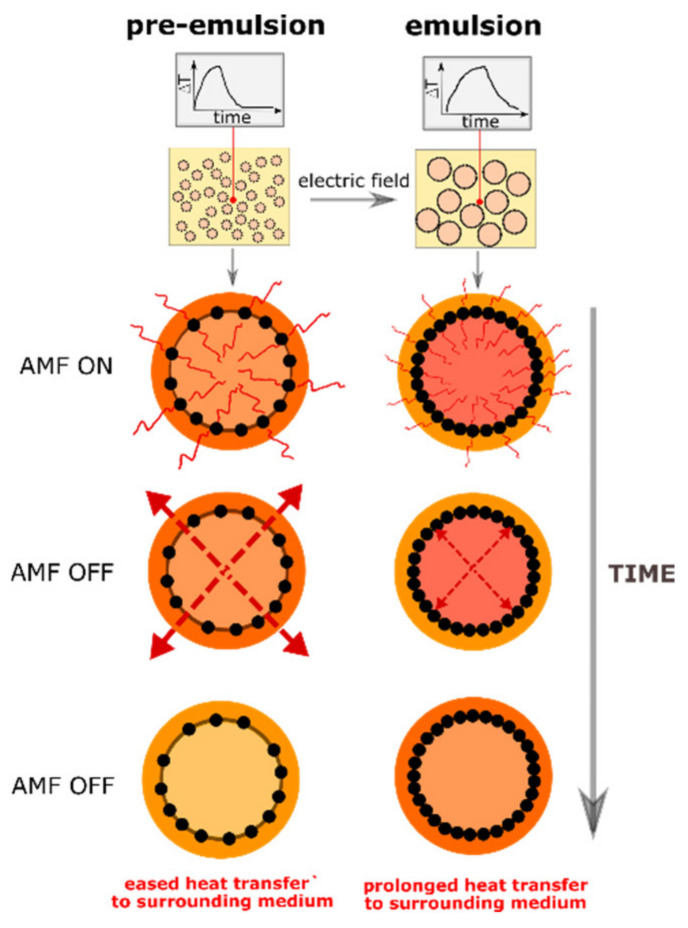
A schematic illustration of the idea of experiments. Different colors relate to different temperatures within the magnetic particle shell and outside this. When the alternating magnetic field (AMF) is on, the red lines represent heat generated by particle excitation both toward inside and outside the droplet. When the AMF is switched off, the red arrows represent heat transfer from the particle shell to the surrounding medium. For a solid particle shell, the heat transfer might be retarded, and in the emulsion the higher temperature is maintained longer.

The heat generation under the AC magnetic field is due to the relaxation and hysteresis losses occurring in magnetic particles and is influenced by several factors such as particle size, intensity of the magnetic field used, viscosity of the hosting medium. In our previous work [20], we showed that also a specific arrangement of particles at the oil-oil interfaces may change the heating performance of emulsions. This change in our experiments is caused by electro-coalescence of emulsion droplets. As the total surface of the dispersed phase decreases when droplets merge, the coverage of droplets increases. This difference can also change the heat transfer after magnetic heating. The postulated differences between pre-emulsions and emulsions that influence the process of heat transfer are illustrated in Figure 2. They may lead to the differences in results from calorimetric measurements when not only the temperature increased but also the cooling down is studied.

In Figure 2, when the AC magnetic field (AMF) is on, the temperature around the droplets is different for the emulsion system with non-solid and more solid particle shells around droplets [20]. In both cases, the heat generated in magnetic particles is transferred into the droplet inside and from the droplet’s surface, as shown schematically with red lines of various lengths. After turning off the magnetic field, the macroscopic temperature in the medium decreases, but in different way due to the varying difficulty of heat transfer from the inside of droplets to the surrounding medium for the droplets in pre-emulsions and emulsions. This hypothesis is explained in the next paragraphs.

### 2.4. Calorimetric Measurements under an AC Magnetic Field

**Figure 3:**
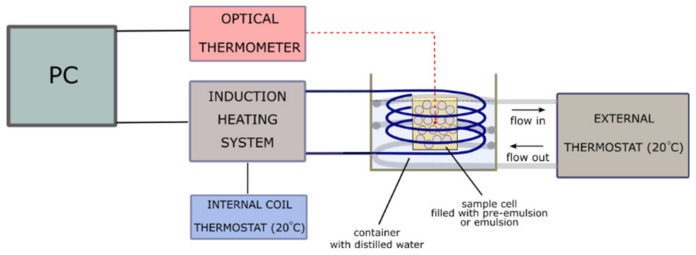
The scheme of the experimental setup for calorimetric measurements of magnetite-stabilized emulsions under an AC magnetic field. The measurement cell filled with either pre-emulsion or emulsion was placed in the induction coil. The induction coil was immersed in the thermostated container with distilled water where the temperature of 20 °C was maintained by an external thermostat. Additionally, the induction was water-cooled to efficiently diminish the effect of heating of the coil.

To evaluate the heating and cooling rates when magnetic emulsion systems were exposed to the alternating magnetic field, a compact induction heating system (EASYHEAT, Ambrell Co., Rochester, NY, USA) was used. The sample cell, filled with either pre-emulsion or emulsion, was placed inside the container with distilled water. The temperature of the water was maintained at 20 °C using an external thermostat. This allowed us to provide constant experimental conditions for all of the measurements regardless of the room temperature. The induction coil was immersed in water, and the sample cell was located in the middle of the coil. The temperature change during magnetic heating was measured by a temperature sensor system (FLUOROTEMP, Photon Control, Burnaby, BC, Canada) equipped with an optic fiber probe (FTP-NY2, ) and placed centrally in the cuvette. A single measurement lasted 600 s. The scheme of the setup for calorimetric measurements is presented in Figure 3.

## 3. Results

### 3.1. Temperature Increase and Decrease in Pre-Emulsions and Stable Emulsions under an AC Magnetic Field

In our experiments, we were able to regulate the dynamics of magnetic heating and to investigate its influence on the process of subsequent cooling. The dynamics were affected by the type of magnetic particles and the intensity of the magnetic field, i.e., the size of particles influences the heating efficiency owing to the increasing effect of hysteresis losses on the overall heat generation. The increased temperature elevation when bigger particles were stabilizers occurred both for merely stable and stable emulsion systems. To show the results to be independent of the dynamics of heating, we conducted another experiment (scenario A) where the time of heating was 30 s. There were no limits in maximum temperature. The different dynamics of heating are in turn clear when comparing the time in which the final temperature was reached. In this experimental scenario (scenario B), the final temperature realizable in the sample was fixed at 25 °C. Above this temperature, the heating system turned off automatically.

**Figure 4:**
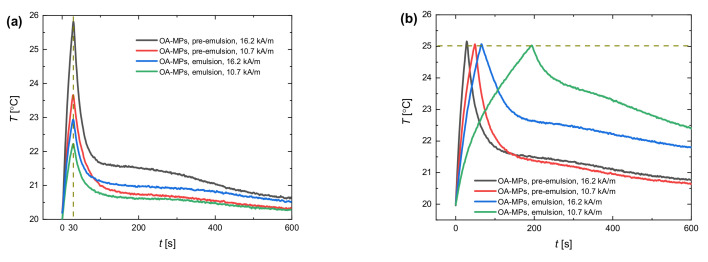
The temperature versus time for measurements under the AC magnetic field of the amplitude of 10.7 and 16.2 kA/m for pre-emulsions and stable emulsions stabilized with OA-MPs for the situation (**a**) when the induction heating system was turned off automatically after 30 s and (**b**) when the induction heating system was turned off automatically when the temperature was above 25 °C. The curves represent the process of heating and the subsequent cooling down process due to the constant temperature in the thermostated container (20 °C) after turning off the magnetic field. The mass concentration of silicone oil in relation to castor oil and the concentration of magnetite were the same for each of samples (10% and 2.3%, respectively). The results had uncertainty values of 1–5% that were not presented in the graph for better clarity.

The comparison between the two scenarios is presented in Figure 4. The one type of magnetic particles—OA-MPs, nanoparticles coated by oleic acid—was chosen as a stabilizer.

As one can see, the temperature evolution in time differed for various magnetic field intensity values and the level of particle shell solidity (pre-emulsions vs. emulsions). In Figure 4a, the highest temperature increase after 30 s of heating was obtained for pre-emulsions under the magnetic field with an intensity of 16.2 kA/m. The fact that pre-emulsions exhibit better heating performance than emulsions was observed and discussed in our previous work [20]. For particles with a small magnetic core such as OA-MPs, the potential reason can be the inhibition of Brown relaxation when residing at the droplet interface. A lower intensity of the magnetic field (10.7 kA/m) led to a significantly smaller temperature increase. When the temperature was limited, the most dynamic temperature increase was achieved for pre-emulsions heated under the AC magnetic field with an intensity of 16.2 kA/m. For lower magnetic field intensity, the temperature elevation was significantly slower.

Interestingly, the temperature in samples decreased not always with the same dynamics, despite the same temperature being maintained in the thermostated container where the induction coil with samples was immersed (20 °C) and the same temperature was reached in the samples (25 °C). From Figure 4 it is clear that the chosen scenario not only influenced the temperature increase but also the way the sample is cooled down. In the next paragraph, we will take a closer look at only the process of cooling for systems stabilized with different magnetic particles.

### 3.2. Cooling Process for Constant Time of Magnetic Heating

**Figure 5:**
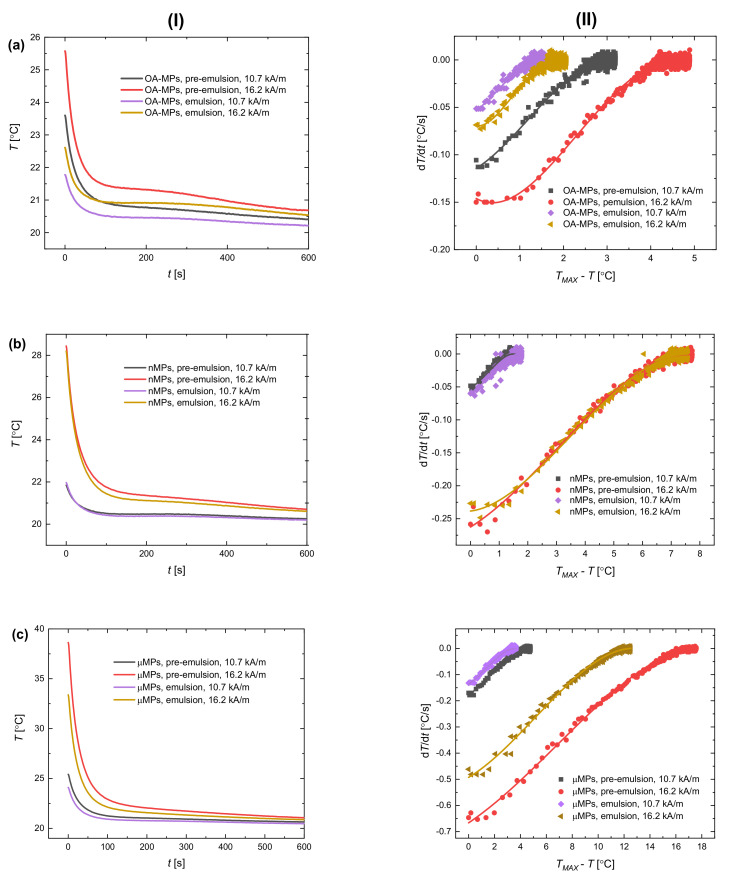
(**I**) The temperature versus time during cooling down after heating and (**II**) the temperature change rate versus temperature difference after magnetic heating for pre- and emulsions stabilized with (**a**) OA-MPs, (**b**) nMPs, and (**c**) µMPs particles for measurements under the AC magnetic field of an amplitude of 10.7 and 16.2 kA/m. The experimental points representing d*T*/d*t* (recorded every second) were fitted to polynomial from Equation (2) whose trending lines were added. The mass concentration of silicone oil in relation to castor oil and the concentration of magnetite were the same for each of samples (10% and 2.3%, respectively), and the operating time of the AC magnetic field was 30 s. The results had uncertainty values of 1–5% that were not presented in the graph for better clarity.

In the first experimental scenario (scenario A), the magnetic field was switched off after 30 s. Figure 5 presents the temporal evolution of the temperature after the AC magnetic field was off (panel I) and the temperature change rate (d*T*/d*t*) plotted as a function of the temperature change between the maximum temperature *T_MAX_* reached during the heating process and the temperature observed for the time after magnetic heating. The analysis of the cooling rate was performed not only qualitatively but also quantitatively. In our case, the use of Newton’s law (see Equation (1)) for fitting the experimental results of temperature decrease was not satisfactory. The coefficient of determination, *R^2^* was in the order of 0.5–0.6, which is clear evidence of a poor agreement between Newton’s equation and the way our system cools down. This is why we utilized the analysis proposed in works of Wildeboer and Lahiri and their co-workers [18,19] based on the Equation (2) to analytically describe the differences in cooling rates between samples.

The results from Figure 5 show the differences in the cooling dynamics when various particles and intensities of the magnetic field were used. The absolute temperature difference after 600 s of measurement is most significant for the systems stabilized with µMPs and higher magnetic field intensity. The temperature change during cooling down is simply dependent on the heating efficiency of a given sample. For samples where the temperature increased more during magnetic heating, the temperature maintains at a higher level after the AC magnetic field is off. That is why the curves representing pre-emulsions with µMPs (Figure 5c) and higher magnetic field intensities are in general higher than others. At the same time, there are also subtle differences between pre-emulsions and emulsions for each sample, i.e., the temperature after dynamic decrease maintains higher values for pre-emulsions than for emulsions. This is the other evidence of the difference in heating efficiency resulting from the change in particle coverage of the droplets.

The results from Figure 5 (panel II) reflect the cooling dynamics and show that is highest just after the magnetic field is turned off. The temperature decreases, so the derivative d*T*/d*t* is negative, and we obtained the lowest values of d*T*/d*t* for temperatures nearest the maximum temperature recorded in the sample, i.e., the smallest values of *T_MAX_* − *T*. The experimental points were successfully fitted to Equation (2) (*R*^2^ = 0.99), which indicates that thermal conduction, convection, and radiation occurred together during the process of cooling our systems for a wide range of temperature increase. As we mentioned, fitting the curves showing temperature decrease vs. time to Equation (1) was not satisfactory, which possibly indicates the strong influence of convection and radiation on the process of cooling in our experiments [24]. Although the differences in the temperature between the surrounding medium (20 °C) and the sample did not exceed a few degrees Celsius, in the case of our emulsion systems, the linear approximation of the results did not bring a desired agreement.

### 3.3. Cooling Process for Constant Maximum Temperature

**Figure 6:**
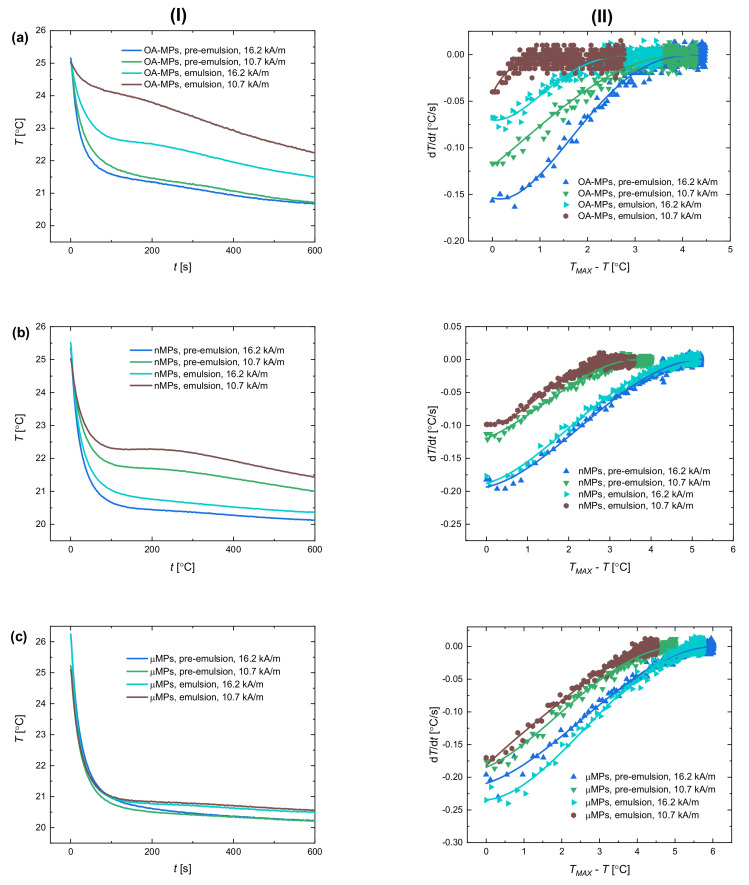
(**I**) The temperature versus time during cooling down after heating and (**II**) the temperature change rate versus temperature difference after magnetic heating for pre- and emulsions stabilized with (**a**) OA-MPs, (**b**) nMPs, and (**c**) µMPs particles for measurements under the AC magnetic field of an amplitude of 10.7 and 16.2 kA/m. The experimental points representing d*T*/d*t* (recorded every second) were fitted to polynomial from Equation (2) whose trending lines were added. The mass concentration of silicone oil in relation to castor oil and the concentration of magnetite were the same for each of samples (10% and 2.3%, respectively) and the time when the AC magnetic field was turned on was when the temperature in the sample exceeded 25 °C. The results had uncertainty values of 1–5% that were not presented in the graph for better clarity.

Much more interesting to understand is how the presence of a stable particle shell around the droplets in Pickering emulsions influences the way heat is transferred is the situation when the samples reach the same fixed temperature (25 °C) in each measurement, as is the case for the experimental scenario B. In this case, the potential differences in cooling between samples cannot be caused simply by the different temperature increase during magnetic heating. The results of the temperature evolution in time after the AC magnetic field was off (panel I) and the calculated values of d*T*/d*t* as a function of the temperature change (panel II) are presented in Figure 6.

Figure 6 shows the reversion in temperature decrease (panel I) for intensities of the magnetic field compared to Figure 5. The samples placed in the magnetic field of lower intensity (10.7 kA/m) needed significantly more time to reach the temperature of 25 °C than samples heated in the AC magnetic field of higher intensity. This difference influenced the way the samples cooled down. As presented, this process is much slower for lower intensities, especially when OA-MPs and nMPs were stabilizers.

One can also see that the process of cooling was evidently less dynamic for emulsions compared to pre-emulsions regardless of the particles used as stabilizers and the intensity of the AC magnetic field. Our explanation is that the heat transfer is indeed retarded for emulsions where droplets are coated by a complete particle shell that influences the temperature measured in the whole sample. The effect of particle coating seems to be less significant for µMPs (Figure 6c), which can be explained by a much higher heating dynamics and the subsequent effective process of transferring heat from the particle shell into the surrounding medium. The abovementioned differences are also reflected in the results of the temperature change rate versus temperature difference (Figure 6, panel II). It is significant that the temperature change rate takes lower values for pre-emulsions than for emulsions. The dynamics of how these values of the experimental points change are also different for various samples.

## 4. Discussion

As we presented above, the process of cooling after magnetic heating in emulsions strongly depended on the factors affecting the heating, i.e., the magnitude of the magnetic field applied, the size of particles, and the time of application of the magnetic field. Therefore, it is possible to adjust parameters such as particle size so that the energy can be stored inside the Pickering droplets for a while. The measured temperature decrease in systems with droplets with stable particle coating can be, under certain conditions, slower compared to a situation where droplets are coated by particles to a less extent. Then, the energy transfer is sustained. From the results (Figure 6), for this purpose it would be better to use the particles with lower heating efficiency.

When the magnetic field is turned off, particles are no longer the sources of heat. Nonetheless, the maximum temperature measured for scenario A exceeded the limit of 25 °C, especially for emulsions stabilized with µMPs (see, the temperature for 0 s in Figure 6c, panel I). This could stem from the fact that energy is entrapped inside the droplet for a while and heat transfer is halted to some extent. It is worth noting that we did not observe this effect in ferrofluids, although the sources of heat and the experimental equipment were the same. For magnetic particles suspended in either castor or silicone oil, the temperature did not exceed that limit, which can be somehow more evidence for the ‘energy capsulation’ occurring in armored droplets. Potentially, the heat entrapped within the droplet could be transported and released under external stimuli in the destined place.

In our paper, we considered macroscopic consequences of nano- and micro-heating from magnetic particles. Nevertheless, the local temperature increase should be high enough to cause a significant temperature increase in the surrounding medium. Additionally, although recent reports suggested that it is not certain [25,26], our results confirm that the particles influence the heating and cooling processes when assessing them in micro- and/or nano-scale.

## 5. Conclusions

In this work, we evaluated the process of cooling in emulsion systems stabilized with magnetic particles. The way our samples cooled down depended strongly on the intensity of the AC magnetic field used for heating, on the size of magnetic particles and also on the solidity of the particle shell around Pickering droplets. The emulsions with droplets coated to a higher extent by magnetic particles have already been reported to exhibit weaker heating performance [20]. However, as shown in this work, they also cooled down slower compared to emulsions with poorly coated droplets. It was especially clear when the comparison between different samples was performed for the same maximum temperature. The results can be the evidence that emulsions with stable magnetic Pickering droplets are good candidates as materials with sustained heat transfer. This could be another application of oil-in-oil emulsions that have numerous advantages but are still understudied [27].

## Figures and Tables

**Table 1:** List of physical parameters of materials used in the experiments. The values were given for room temperature. If not otherwise stated, the values were taken from data sheets.

	Castor Oil	Silicone Oil	Magnetite Particles
Dynamic viscosity [mPa·s]	700	50	-
Thermal conductivity [W/m·K]	0.18	0.15	5.0 [21]
Specific heat [J/kg·K]	1800	1460	950 (OA-MPs, calculated ^1^) 650 (pristine MPs, [22])
Magnetization saturation [emu/g]	-	-	63.67 (OA-MPs, measured, [20]) 89.46 (nMPs, measured, [20]) 85.06 (µMPs, measured, [20])

^1^ In the case of magnetic particles functionalized with oleic acid (OA-MPs), we assumed that the oleic coating accounts for 20% of the mass of magnetic material. The weighted value of specific heat was calculated as in [22].
